# Modulation of the modulated magnetic structure of an Ho i-MAX phase described by a magnetic (3+2)-dimensional superspace group

**DOI:** 10.1107/S2052520624011053

**Published:** 2025-01-23

**Authors:** Claire V. Colin, Quanzheng Tao, Christine Opagiste, Rafik Balou, Johanna Rosen, Thierry Ouisse, Václav Petříček

**Affiliations:** a Institut Néel Université Grenoble Alpes & CNRS Grenoble 38042 France; b Department of Physics, Chemistry and Biology (IFM) Linkopings Universitet Linköping SE-58183 Sweden; c Université Grenoble Alpes Centre national de la recherche scientifique (CNRS), Grenoble INP, Laboratoire des Matériaux et du Génie Physique (LMGP) Grenoble 38000 France; d Institute of Physics of the Czech Academy of Sciences Na Slovance 1999/2 18200Praha 8 Czechia; Universidad del País Vasco, Spain

**Keywords:** magnetic superspace groups, incommensurate structures, rare-earth i-MAX phases, neutron diffraction

## Abstract

The magnetic structures of the Ho-based i-MAX phase (Mo_2/3_Ho_1/3_)_2_GaC were studied with neutron powder diffraction at low temperature. (Mo_2/3_Ho_1/3_)_2_GaC crystallizes in the ortho­rhombic space group *Cmcm*. The material undergoes two successive antiferromagnetic transitions at *T*_N1_ = 10 K and *T*_N2_ = 7.2 K. The magnetic structure below *T*_N1_ is incommensurate with the propagation vector **k**_1_ = (0, *k_y_*, 0) with *k_y_* = 0.696 (1) at 9 K. For the analysis of the magnetic structure, a group-theoretical approach based on the space group of the nuclear structure and its subgroups was employed. A model in the (3+1)D superspace group *Cmcm*.1′(0β0)*s*0*ss* yielded the most accurate results in neutron powder diffraction refinements. The determined structure was found to be an incommensurate longitudinal amplitude-modulated magnetic structure. Below *T*_N2_, additional magnetic satellites develop. They could be indexed by a propagation vector **k**_2_ = (τ_*x*_, 0, 0) with the τ_*x*_ value increasing below *T*_N2_ until it stabilizes at approximately 3 K at 0.075. A magnetic structure determination considering two propagation vectors **k**_1_ and **k**_2_ was carried out using the superspace formalism by building the corresponding (3+2)D model. The determination was based on the observation that the additional magnetic peaks emerge exclusively in the vicinity of the incommensurate magnetic peaks with propagation vector **k**_1_, and not in the vicinity of nuclear peaks. This indicates that only mixed-index reflections were observed, and not reflections purely related to **k**_2_. The magnetic superspace group (MSSG) that was determined is *Amma*.1′ (0,β,0)00*s*0 (0,0,γ)*ss*0*s*. The structure can be described as a longitudinal amplitude-modulated structure, which itself is amplitude-modulated in a perpendicular direction. This represents a very unusual case of a 2-**k** magnetic structure with no symmetry relation between the propagation vectors.

## Abstract 2

A rare complex incommensurate magnetic structure, an amplitude-modulated structure which itself is modulated, was determined in the Ho-based i-MAX phase (Mo_2/3_Ho_1/3_)_2_GaC. It represents a particularly distinctive case of a 2-**k** magnetic structure with no symmetry relation between the propagation vectors.

## 1. Introduction

In contrast to incommensurately modulated crystal structures, where the superspace approach is generally recognized as the standard method, the solution and refinement of magnetic structures were, until recently, typically carried out using a different approach. This involved decomposing the magnetic configuration space into basis modes that transform according to the physically irreducible representations (henceforth irreps) of the space of the paramagnetic space group, so-called representation analysis (Bertaut, 1968 ▸). It was only recently that it was recognized that the direct use of Shubnikov space and superspace groups facilitate work with non-modulated and modulated magnetic structures (Petríček *et al.*, 2010 ▸). The relevance of superspace for the study of magnetically ordered structures is demonstrated by the complete determination of the global symmetry of the system (in terms of magnetic and nuclear contributions) expressed with crystallographic rules (Stokes & Campbell, 2022 ▸; Perez-Mato *et al.*, 2012 ▸). The use of magnetic superspace symmetry ensures a robust and unambiguous description of both atomic positions and magnetic moments within a common unique formalism. In recent years, the joint development of group-theory tools such as the *ISODISTORT Software Suite* (Campbell *et al.*, 2006 ▸; https://iso.byu.edu) as well as refinement software such as *Jana2006* (Petříček *et al.*, 2014 ▸) and *Jana2020* (Petříček *et al.*, 2023 ▸) have provided essential tools to extend the application of the superspace formalism to magnetic structure analysis. A review of the MAGNDATA database (Gallego *et al.*, 2016 ▸) reveals that 150 incommensurate magnetic structures with one propagation vector have been included in the database to date, but only two instances with two propagation vectors and one instance of three. In this paper, we illustrate the successful application of superspace formalism to the determination and analysis of the complex low-temperature magnetic structures of the Ho based i-MAX phase (Mo_2/3_Ho_1/3_)_2_GaC with two propagation vectors.

**Figure 1:**
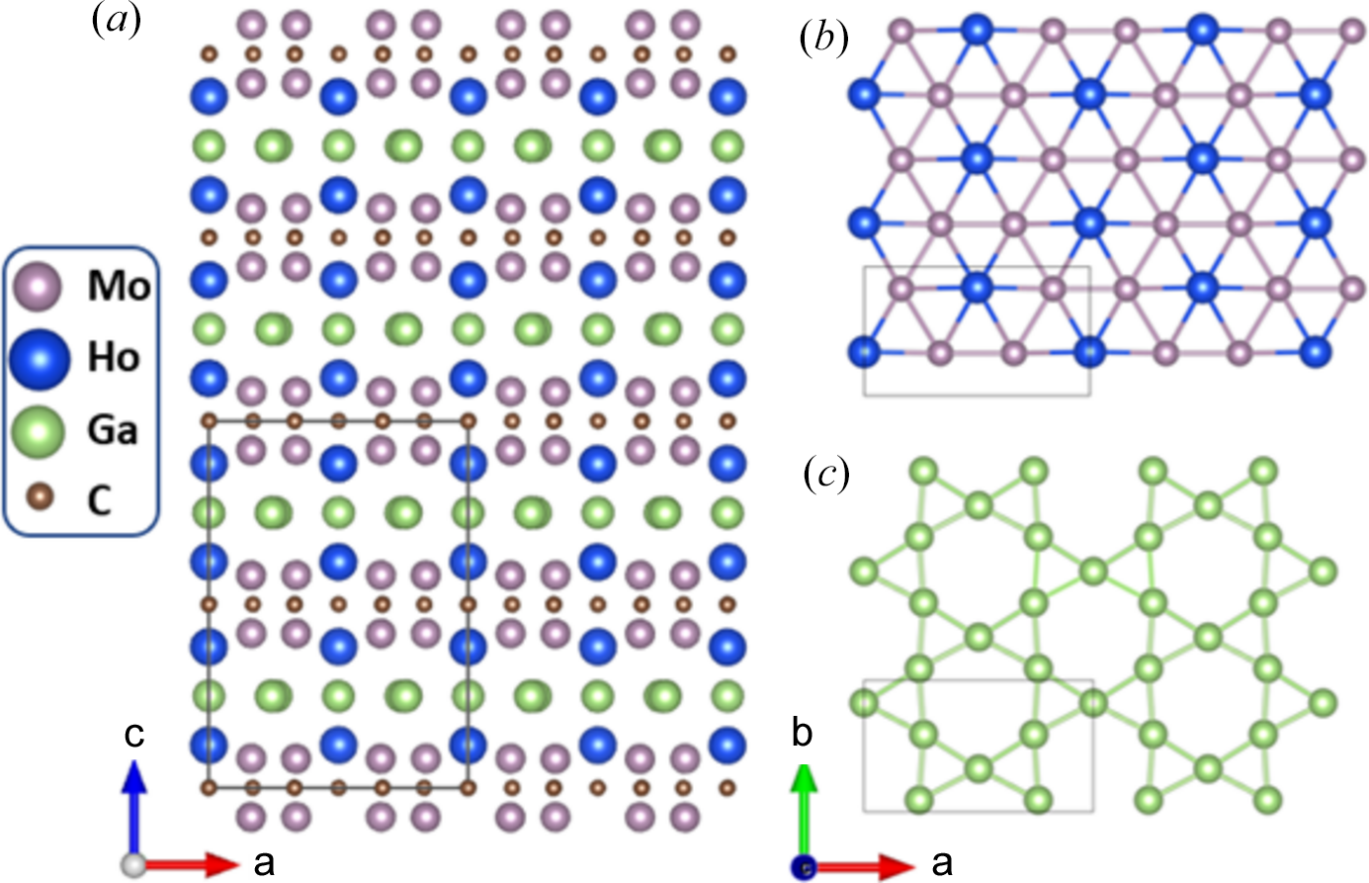
Projection of the orthorhombic crystal structure of (Mo_2/3_Ho_1/3_)_2_GaC along the *b* axis (*a*), with corresponding top view of the in-plane ordered Mo_2/3_Ho_1/3_ layer consisting of a triangular Ho lattice overlaid with a honeycomb arrangement of Mo (*b*) and a Ga kagome layer (*c*).

In-plane ordered MAX phases (Dahlqvist *et al.*, 2017 ▸), also known as i-MAX phases, are derived from layered hexagonal MAX structures (Barsoum, 2000 ▸). In 2019, it was discovered that these quaternary compounds of general formula (*M*^1^_2/3_*M*^2^_1/3_)_2_*AX* can accommodate rare-earth (RE) atoms on the *M*^2^ site, resulting in magnetic properties. To date, two families of rare-earth i-MAX phases have been reported: (Mo_2/3_RE_1/3_)_2_AlC (Tao *et al.*, 2019 ▸) and (Mo_2/3_RE_1/3_)_2_GaC (Petruhins *et al.*, 2019 ▸). The layered structure of these phases comprises a stack of Mo_2/3_RE_1/3_ layers sandwiching a C layer, separated by an *A* kagome plane (Fig. 1 ▸). The RE ions, which constitute one-third of the atoms in the layers, are ordered in a skewed triangular lattice with a distance close to 5.5 Å. They then form quasi-2D magnetic triangular lattice bilayers with similar intralayer and interlayer distances (3.65 Å and 3.63 Å, respectively). The very localized and strongly magnetic 4*f* orbitals interact through the oscillating Ruderman–Kittel–Kasuya–Yosida (RKKY) coupling, leading to a magnetic order. The competition between oscillating couplings and the configuration of the RE network can lead to frustration of the magnetic moments. The crystal electric field (CEF) acts on the aspherical 4*f* orbitals, resulting in magneto-crystalline anisotropy. Furthermore, the strong structural anisotropy is likely to impact the physical properties. The magnetic and electronic behaviour of these compounds is, therefore, the result of a delicate balance between numerous contributions, leading to ground states that are strongly dependent on the RE element and can evolve rapidly when the compound is subjected to an applied magnetic field or if the temperature is modified. This phenomenon has been observed in several systems belonging to the (Mo_2/3_RE_1/3_)_2_AlC family, as shown by powder neutron diffraction studies conducted by Tao *et al.* (2019 ▸) and Potashnikov *et al.* (2021 ▸). More recently, single-crystal neutron diffraction under a magnetic field has also provided evidence of this phenomenon (Barbier *et al.*, 2022 ▸).

In this study, we present the determination of complex magnetic structures in the Ho-based i-MAX phase (Mo_2/3_Ho_1/3_)_2_GaC derived from neutron powder diffraction. To this end, we employ two different magnetic models within the superspace formalism, one being (3+1)D and the other (3+2)D.

## 2. Experimental

Polycrystalline (Mo_2/3_Ho_1/3_)_2_GaC was synthesized by solid-state reaction of elemental powders of graphite, Mo, Ga and Ho. Mo, Ho, Ga and C were mixed in a stoichiometric ratio of 4:2:3:3. First, the Mo, Ho and C powders were mechanically mixed in an agate mortar. The powder mixture was then placed in an alumina crucible, Ga pellets were added and the pellet/powder mixture was stirred. The alumina crucible with its contents was then heated under an Ar flow to 1400°C at a rate of 10°C min^−1^ and then held at 1400°C for 5 h. The loosely sintered powders were crushed into a fine powder that was directly used for further analysis.

Bulk magnetization measurements were conducted utilizing a commercial Quantum Design MPMS magnetometer across a temperature range of 2–300 K, with an applied magnetic field of up to 7 T. Specific heat measurements (*C*_p_) were obtained via the relaxation method with a Quantum Design PPMS across a temperature range of 2–300 K and under magnetic fields of up to 5 T. The transition temperature was determined from the inflexion point of each lambda anomaly.

Neutron powder diffraction (NPD) measurements were carried out using the CRG-D1B high-flux powder diffractometer at the Institut Laue–Langevin (Ouisse & Colin, 2018 ▸). Approximately 1 g of a powdered sample of (Mo_2/3_Ho_1/3_)_2_GaC was loaded into a vanadium sample holder. Measurements were conducted using a vertically focusing HOPG monochromator to produce a neutron wavelength of λ= 2.526 Å, and a Ge monochromator to produce a neutron wavelength of λ = 1.285 Å. The data were collected using a ^3^He detector bank covering a 128° 2θ range in steps of 0.1°.

## 3. Results

### 3.1. Crystallographic structure

The Ho based i-MAX phase (Mo_2/3_Ho_1/3_)_2_GaC crystallizes in the orthorhombic space group *Cmcm* (No. 63) with the structure depicted in Fig. 1 ▸. Rietveld refinement of NPD data confirms the structure already reported (Petruhins *et al.*, 2019 ▸), see Fig. S1 and Table S1 in the supporting information. The polycrystalline sample contains impurities, some of which are magnetic, such as HoGa_3_ and Ho_2_O_3_. The weight fraction of the impurities is provided in Fig. S1 for reference.

### 3.2. Bulk magnetic characterization

**Figure 2:**
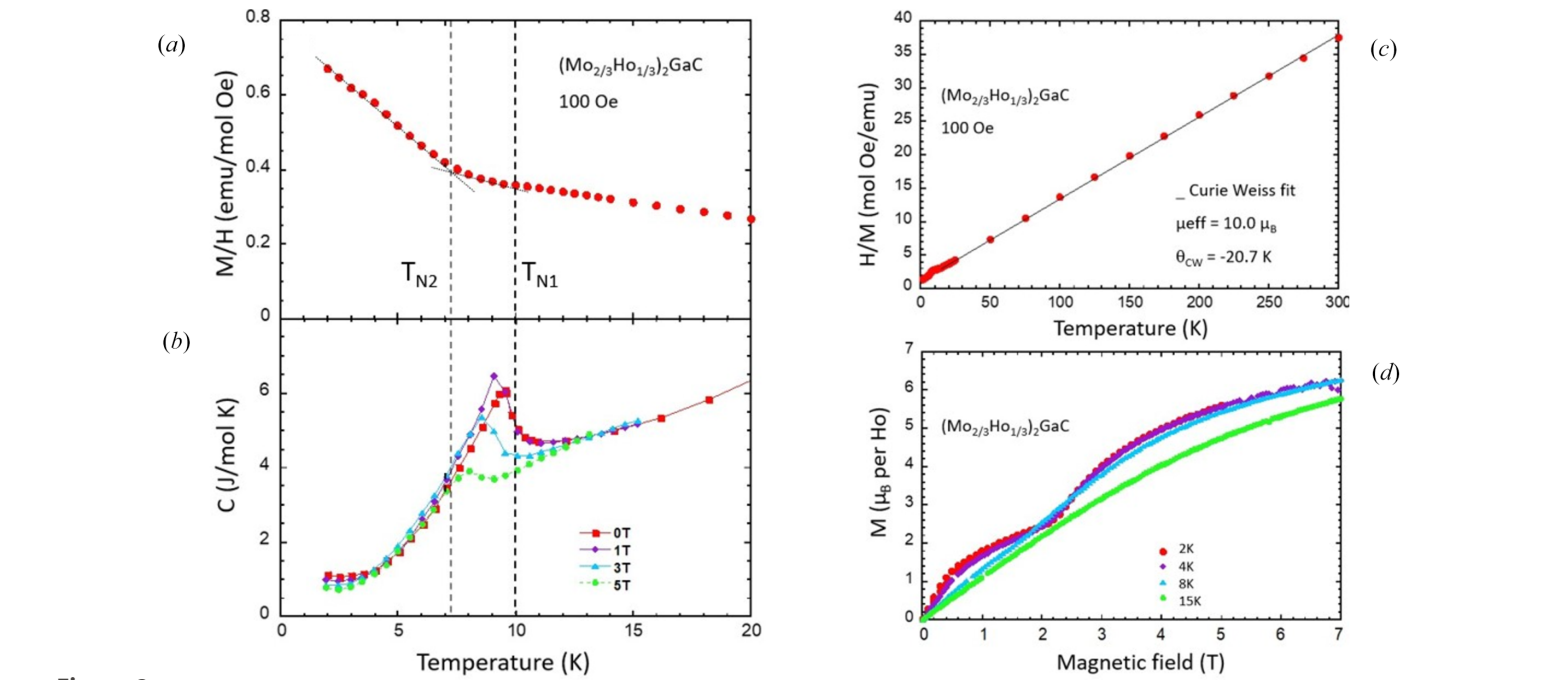
Magnetization and specific heat measurements carried out on a powder sample of (Mo_2/3_Ho_1/3_)_2_GaC. (*a*) Magnetic susceptibility recorded at 100 Oe and (*b*) specific heat as a function of temperature below 20 K. (*c*) Temperature dependence of inverse magnetic susceptibility between 2 and 300 K. (*d*) Magnetization versus field at different temperatures.

Bulk magnetization and specific heat measurements were carried out on a powder sample of (Mo_2/3_Ho_1/3_)_2_GaC. Fig. 2 ▸(*a*) shows the magnetic susceptibility versus temperature curve recorded at low magnetic field (100 Oe). A kink and an inflection can be observed in the curve centered on 7.2 (3) K and 10.0 (3) K, respectively, marked as *T*_N2_ and *T*_N1_. Fig. 2 ▸(*b*) illustrates the temperature dependence of *C*_p_ in different applied magnetic fields, ranging from 0 T up to 5 T. In the absence of an applied magnetic field, the onset of the antiferromagnetic long-range ordering is clearly shown by a lambda anomaly at 10.0 (2) K, which corresponds to the inflexion point observed in the magnetic susceptibility measurements (marked as *T*_N1_). Below 4 K, a slight increase in specific heat is observed, which can be attributed to the nuclear hyperfine contribution in holmium (Gordon *et al.*, 1961 ▸). This phenomenon has already been observed in numerous holmium-based compounds and also in the Ho i-MAX parent phase (Barbier *et al.*, 2022 ▸).

The Curie–Weiss fit performed between 50 and 300 K on the inverse of the susceptibility in Fig. 2 ▸(*c*) yielded an effective moment of 10 μ_B_, which is consistent with the expected value of 10.6 μ_B_ for holmium. The Weiss temperature, which is negative, −20.7 K, indicates that the interactions are predominantly antiferromagnetic. Fig. 2 ▸(*d*) shows the magnetization versus field at different temperatures. The field behaviour changes drastically with temperature. In the intermediate temperature range, *i.e.* below *T*_N1_ and above *T*_N2_, no field-induced transition is visible. However, at low temperatures (below *T*_N2_), a metamagnetic transition is observed around 2.3 T. It is important to note that, regardless of temperature, no remanent magnetization was observed at 0 T, and saturation of the Ho moment was not reached for the strongest measured fields (only 6 μ_B_ per Ho at 7 T).

### 3.3. Temperature dependence of the neutron powder diffraction

**Figure 3:**
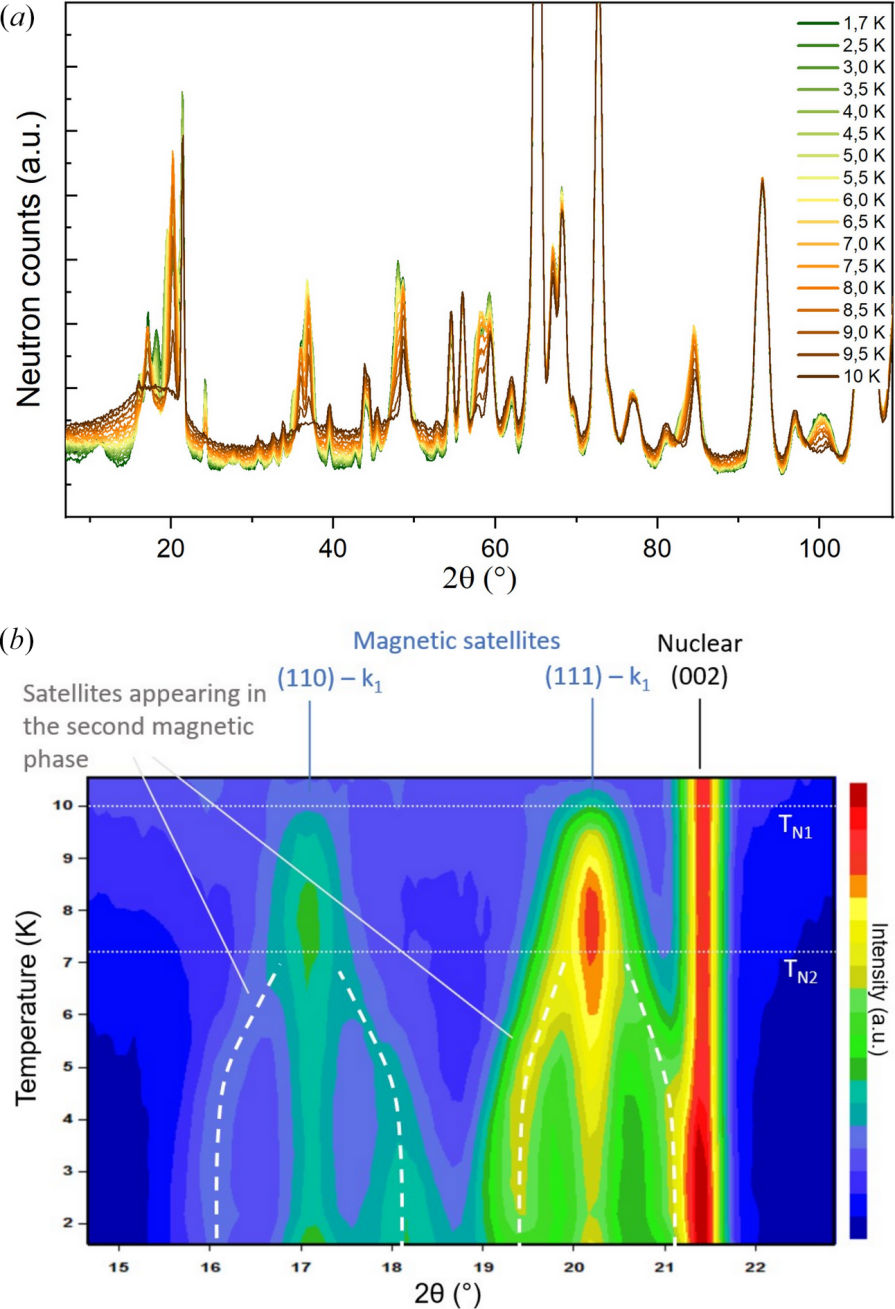
(*a*) Temperature dependence of neutron powder diffraction patterns recorded at 2.52 Å between 1.7 and 10 K. (*b*) 2D map of the low-angle part of the temperature dependence of the diffraction patterns, showing the strongest magnetic lines and evidencing the two magnetic ordering transitions.

Fig. 3 ▸ illustrates the temperature dependence of the neutron powder diffraction patterns. An additional magnetic contribution and a drop of the paramagnetic contribution of the background is clearly visible below *T*_N1_. As shown in the low-angle diffraction patterns presented in panel (*b*) as a 2D map, there are two stages in the magnetic ordering process. Below *T*_N1_, a series of satellite peaks emerges. All of the observed peaks can be indexed with a single incommensurate **k**-vector along the *b* direction, **k**_1_ = (0, *k_y_*, 0). Below *T*_N2_, a new series of peaks appears. As can be observed in Fig. 3 ▸(*b*), the additional magnetic peaks emerge exclusively in the vicinity of the incommensurate magnetic peaks indexed with propagation vector **k**_1_, and not in the vicinity of nuclear peaks. They can be thought of as the magnetic satellites of the magnetic satellites.

### 3.4. Magnetic structure determination of the intermediate-temperature magnetic phase

**Figure 4:**
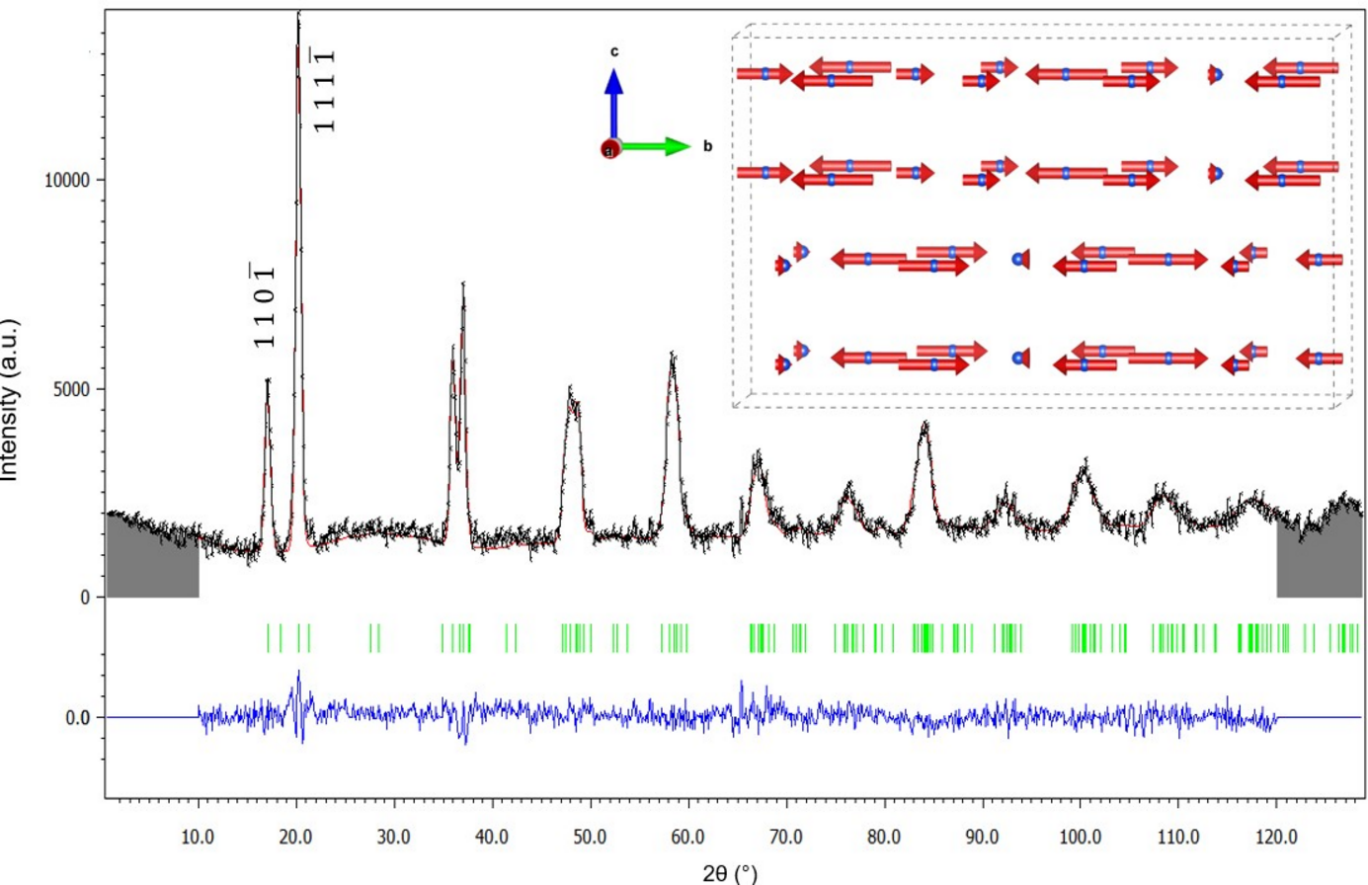
Magnetic structure refinement of neutron magnetic scattering of (Mo_2/3_Ho_1/3_)_2_GaC at 9 K. Observed intensity (black), calculated (red), difference (blue). Magnetic satellite Bragg positions are indicated by green vertical dashes. Inset: schematic of the incommensurate amplitude-modulated longitudinal structure at 9 K (four unit cells along **b** are displayed). Only Ho magnetic cations are shown for clarity.

It was previously established that the magnetic phase between *T*_N1_ and *T*_N2_ features magnetic peaks that can all be indexed by an incommensurate propagation vector along **b**. The magnetic structure was determined and refined at 9 K, for which **k**_1_ = (0, 0.696 (1), 0). The magnetic space group was determined using the group-theory program *ISODISTORT*, considering the paramagnetic space group *Cmcm.*1′ (No. 63), the **k**_1_ propagation vector symmetry and the holmium magnetic cation *8f* Wyckoff position. The four potential maximum magnetic superspace groups (MSSGs) were subjected to rigorous testing, with only one, *Cmcm*.1′(0β0)*s*0*ss*, demonstrating a satisfactory fit to the data (Fig. 4 ▸). It is important to note that, in order to limit the potential problems associated with impurities in the sample, the refinement of the magnetic structure was made on the magnetic signal only (*i.e.* the diffraction signal at 9 K subtracted from the paramagnetic diffraction signal at 10 K) and atomic positions were kept at those found at 10 K. Symmetry prohibits the presence of any magnetic component along **a**. Refinement shows that the moment is primarily oriented along the *b* axis and constraining it solely in this direction does not significantly impair the fit. This is the reason why this solution with a limited number of parameters was selected. The magnetic structure that was determined is an incommensurate longitudinal amplitude-modulated structure along the *b* axis as depicted in the inset of Fig. 4 ▸, and is referred to as AM1 in the following. Table 1 ▸ presents a detailed description of the structure within the superspace-group formalism.

### 3.5. Magnetic structure determination of the low-temperature magnetic phase

**Figure 5:**
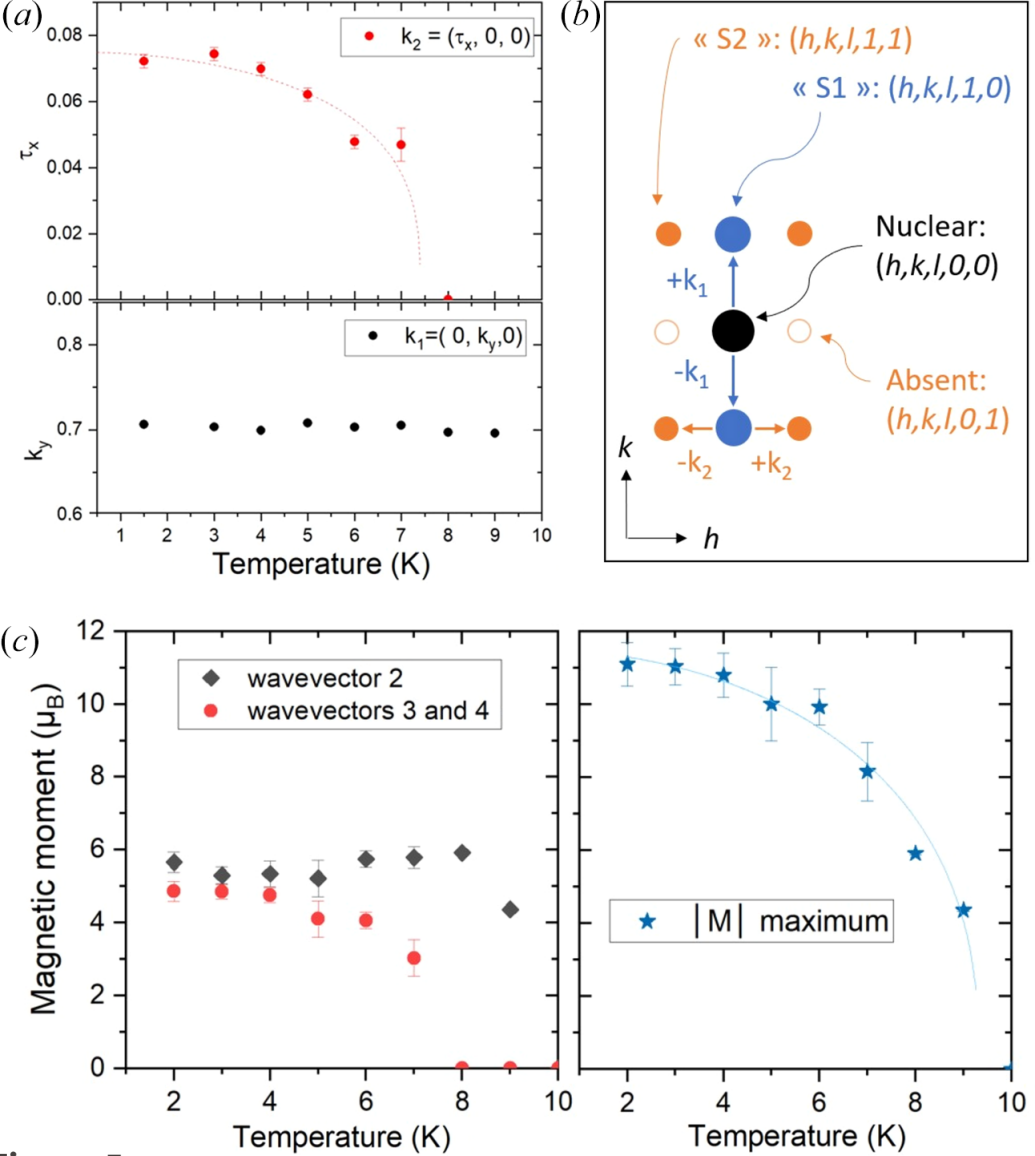
(*a*) Temperature dependence of propagation vectors **k**_1_ and **k**_2_. (*b*) Schematic of the (*hk*0) plane of reciprocal space. Black dots: nuclear peak. Blue dots: magnetic satellites S1. Orange dots: magnetic satellites S2. Open orange dots: satellites extinguished. (*c*) Refined magnetic moment amplitude of wavevectors 2 (corresponding to the S1 satellites and **k**_1_ propagation vector) and 3 and 4 (S2 satellites, corresponding to **k**_1_ + **k**_2_ and **k**_1_ − **k**_2_ propagation vectors) plotted as a function of temperature (see the model presented in Table 2 ▸). The right-hand panel shows the maximum of the absolute value of the magnetic moment of Ho as a function of temperature.

Below *T*_N2_, additional magnetic satellites, designated as S2 in the following text, develop around the incommensurate magnetic peaks indexed by the propagation vector **k**_1_ (satellites S1), as shown in Fig. 5 ▸(*b*). The initial step in solving the structure is to index these S2 satellites. All attempts to index them conventionally with respect to the nuclear lattice were unsuccessful. As satellites S2 can be considered satellites of satellites S1, further attempts were made to index them with reference to the AM1 magnetic incommensurate lattice. To this end, the incommensurable structure was approximated by a pseudo-commensurate unit cell tripled along **b**, assuming *k*_1*y*_ ≃ 2/3. This approach yielded two types of modulations that were in relatively good agreement with the position of the S2 peaks: along **a** or along **b**. A Le Bail refinement indicated that the propagation vector along **a** led to a slightly better description. The refined value of the vector at 3 K is **k**_2_ = (τ_*x*_, 0, 0) with τ_*x*_ = 0.0744 (2). As illustrated in Fig. 5 ▸(*a*), the amplitude of the vector **k**_2_ decreases as the temperature approaches *T*_N2_. In contrast, the vector **k**_1_ remains substantially constant. Consequently, in the following section, the magnetic structure will be determined by considering the combination of two propagation vectors: **k**_1_ = (0, *k_y_*, 0) and **k**_2_ = (τ_*x*_, 0, 0).

Several attempts were made to construct a model with two superimposed magnetic phases. The first phase is the AM1 phase, which orders below *T*_N1_, the second is indexed by a propagation vector (τ_*x*_, *k_y_*, 0) to account for the remaining peak positions. A modulated structure model with moments aligned along **a** and **b** reproduces the data relatively well. Nevertheless, the potential explanations for the presence of two magnetic phases in the sample were deemed to be physically implausible. Firstly, the refinement of the structure revealed no intersite mixing between Ho and Mo, which could have been a mechanism for a large magnetic phase separation due to inhomogeneities in the sample. Secondly, there was no indication in the specific heat of a first-order transition in *T*_N2_ that could have generated phase coexistence. It is therefore unclear whether the actual structure, which is likely to be a single phase, is accurately represented by the sum of the two phases (0, *k_y_*, 0) and (τ_*x*_, *k_y_*, 0). Furthermore, describing and establishing the symmetry of each of these phases separately does not establish the overall symmetry of the system. A unified joint description was missing.

In order to address these issues, we undertook the determination of the structure of a unique phase described by the two propagation vectors in the formalism of (3+2)-dimensional superspace. A trial-and-error search via *ISODISTORT* exploration of (3+2)D MSSGs with (0, *k_y_*, 0) and (τ_*x*_, 0, 0) yielded a vast number of possibilities. Therefore, in order to narrow down the search, it was necessary to constrain it. Two guiding principles were employed in the construction of the model:

(i) Given that the first modulation vector and two of the possibilities for the new vector are compatible with ortho­rhombic symmetry, the first assumption was to construct superspace groups with orthorhombic symmetry by keeping the symmetry operations of AM1 [(3+1)D] and extending it to (3+2)D.

(ii) All diffraction spots can be indexed with five indices with respect to the five vectors a*, b*, c*, **k**_1_, **k**_2_. This implies that from the superspace approach, a set of reflections (*h*, *k*, *l*, *m*, *n*) can be derived. The observation that only new satellites can be observed with respect to old ones can be expressed by the condition that satellite intensities are detected for (*h*, *k*, *l*, *m*, 0), (*h*, *k*, *l*, *m*, *n*) with *m* ≠ 0 and *n* ≠ 0 but not for (*h*, *k*, *l*, 0, *n*) [see Fig. 5 ▸(*b*)]. As demonstrated in Appendix *A*[App appa], this dictates the form of the operator that combines time inversion with a translation in the internal subspace of the superspace.

**Figure 6:**
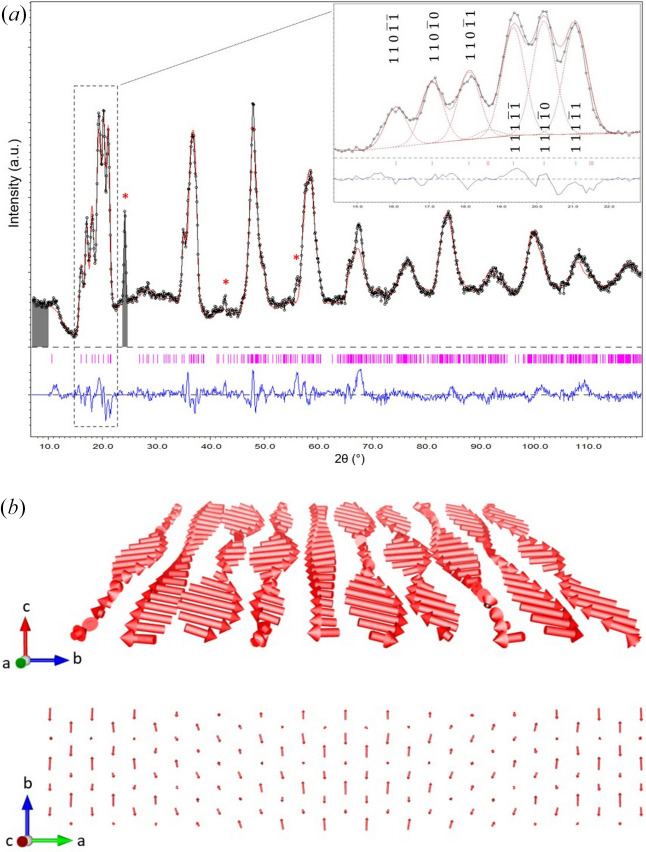
(*a*) Magnetic structure refinement of neutron magnetic scattering of (Mo_2/3_Ho_1/3_)_2_GaC at 3 K. Observed intensity (black), calculated (red) and difference (blue). Magnetic satellite Bragg positions are indicated by purple vertical dashes. The red star symbols indicate the positions of the magnetic peaks of the HoGa_3_ impurity, which is ordered below 8 K. Inset: zoom of the low-angle part (15–22°) of the refinement. Note that the reflection indices are given in the parent phase basis (*Cmcm*). (*b*) Schematic of the incommensurate amplitude-modulated structure at 3 K (*Cmcm* unit-cell axes). Only one plane of Ho magnetic cations is shown for clarity.

Four distinct possibilities were tested with different sets of generators derived from the 4D model in conjunction with the operator constructed with the guiding principle (ii) and an inversion centre. The refinement agreements are presented in Table S2. Two of these models yielded a satisfactory fit to the data, as illustrated in Fig. 6 ▸(*a*). However, of these two models, only one produces magnetic moments with an amplitude close to that theoretically expected for holmium (*g*_*j*_*J* = 10 μ_B_), while the other solution (presented in Table S3) produces considerably larger moments (Fig. S2), which is why it was discarded. The refinement was made on the magnetic signal alone (*i.e.* the diffraction signal at 3 K subtracted from the paramagnetic diffraction signal at 10 K), with the atomic positions remaining as refined at 10 K. The magnetic contribution of an HoGa_3_ impurity is visible below 8 K, indicated by star symbols in Fig. 6 ▸(*a*). The magnetic model comprises ten independent parameters. In order to prevent the occurrence of false minima during the refinement process, a random search for magnetic moments within the models was implemented. Table 2 ▸ presents a detailed description of the structure within the superspace-group formalism in (3+2)D. The crystal structure exhibits orthorhombic symmetry described by the magnetic superspace group symbol *Amma*.1′ (0,β,0)00*s*0 (0,0,γ)*ss*0*s*.

The magnetic structure that was determined is a complex incommensurate amplitude-modulated structure, depicted in Fig. 6 ▸(*b*). Like the AM1 phase, magnetic moments lie predominantly along the *b* direction (*c* in the magnetic *Amma* basis description) and are amplitude modulated along this direction with a fairly short period (8 Å). Along **a** (**b** in the *Amma* basis), the moments are modulated with a much longer period (about 130 Å) with additional components for the magnetic moments mainly along **b** (**c** in the *Amma* basis). They behave like ribbons twisted on themselves along this direction.

Although the proposed model is able to describe the data in a convincing manner, it should be noted that there is a broad magnetic contribution around 2θ = 11° that grows below 3 K which is not explained. It may be the case that this belongs to the magnetic ordering of the Ho_2_O_3_ impurity, which orders antiferromagnetically at a Néel temperature of 2 K.

Using the proposed magnetic model, we refined the temperature dependence. Fig. 5 ▸(*c*) shows the magnetic moment amplitude for wavevectors 2 and 3 (which correspond to **k**_1_ + **k**_2_ and **k**_1_ − **k**_2_ reflections), together with the maximum value of the magnetic moment amplitude as a function of temperature. The maximum value reached by the magnetic moment is close to 11 μ_B_ at 2 K (with an average moment of 5.5 μ_B_). The latter value is slightly higher than the maximum magnetic moment expected for the ground multiplet (*L* = 6, *S* = 2, *J* = 8, 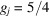) of an Ho^3+^ ion, namely *M* = *g_j_J* = 10 μ_B_. This discrepancy may be due to an underestimation of the scale factor of the magnetic phase, which is determined by the refinement of the nuclear structure at 10 K. This is probably due to the presence of impurities. In addition, there may be some uncertainty in the assessment of the background.

For the sake of completeness, we also considered (3+2)D magnetic models with lower symmetry. As previously stated, all the magnetic peaks can be indexed by considering two modulation vectors: (0, *k_y_*, 0) and (τ_*x*_, *k_y_*, 0). However, this combination is incompatible with the orthorhombic symmetry; in fact the vector (τ_*x*_, *k_y_*, 0) (**k**-plane *P*) lowers the symmetry to a monoclinic *P*2_1_/*m*.1′ symmetry. A systematic investigation is presented in the supporting information. The results obtained for monoclinic models are significantly inferior to those obtained when orthorhombic symmetry is considered. Note that an orthorhombic model can also be constructed by considering three modulation vectors: (0, *k_y_*, 0), (τ_*x*_, *k_y_*, 0) and (−τ_*x*_, *k_y_*, 0), where the value of *k_y_* is identical in the three components. However, the refinements performed with the resulting superspace groups of type (3+3)D gave a fit of significantly lower quality and unphysical moment amplitude (see the supporting information for the results).

### 3.6. Discussion and conclusion

The most common occurrence of magnetic compounds exhibiting more than one propagation vector are the so-called multi-**k** structures, which have been observed in some intermetallic compounds of high crystallographic symmetry (Rossat-Mignod, 1987 ▸). The term ‘multi-**k** structure’ refers to a magnetic structure in which more than one arm of the star of **k** participates in the actual spin arrangement. This phenomenon is observed in topologically non-trivial magnetic structures of the hedgehog and skyrmion type. For instance, the magnetic structure in MnGe (Pomjakushin *et al.*, 2023 ▸) can be cited as a recent illustrative example. The case of two propagation vectors that do not belong to the same star is unusual (Rodríguez-Carvajal & Villain, 2019 ▸), with the exception of conical structures, where the magnetic moments are parallel to the surface of a cone. This corresponds to two propagation vectors **k**_1_ = **k** and **k**_2_ = 0. The conical structure can be obtained by applying an external field on a helical spin configuration, or by the interaction of two spin families, one of which is ferromagnetically ordered and the other is helically ordered. Such structures have been observed, for example, in multiferroic hexaferrites (Qureshi *et al.*, 2018 ▸).

The case of the Ho-based i-MAX phase (Mo_2/3_Ho_1/3_)_2_GaC is notable for its unique characteristics. To our knowledge, it is a rare instance where two incommensurable propagation vectors (not linked by symmetry) are simultaneously present (without phase separation). Another peculiarity is that the modulated structure appears to be modulated itself: the satellites of the second wavevector are only visible around the satellites of the first vector. The magnetic structure develops in two stages. Initially, it undergoes a first transition towards an amplitude-modulated structure. This first magnetic transition is associated with the mDT2 magnetic representation. Subsequently, it undergoes a second transition that modulates it in another direction. This second transition seems to be associated with an irreducible representation SM4, which is by definition ‘even’ for the time-reversal operation. This is an unusual feature, but it is a crucial point that makes this structure completely original. The SM4 modulation would represent a ‘nuclear’ modulation if it was to refer to a nuclear base structure. However, in the present case, the ‘nuclear’ SM4 modulation applies to a magnetic structure. Consequently, its operations are associated with operations that combine time reversal with some other type of operation of the basic magnetic structure, thereby becoming purely magnetic. This is shown by the fact that satellites (0, 1) linked exclusively to the SM4 representation are not detected. Indeed, they would be purely nuclear. On the contrary, mixed reflections (1, 1) and (1, −1) are detected; these reflections are purely magnetic and correspond truly to a magnetic modulation according to wavevectors on the plane *P* of the Brillouin zone. This is why we refer to the low-temperature phase as a modulation of a modulated magnetic structure. The resulting complex structure is remarkably well described using the superspace-group formalism with a limited number of independent parameters. This demonstrates the power of this formalism. However, it is important to keep in mind that even if the magnetic model presented here reasonably explains the data, it cannot be excluded that other models could do the same.

The observation of the amplitude-modulated phase down to the lowest temperature is intriguing, since for well stabilized magnetic moments amplitude modulation would be reflected in residual magnetic fluctuations that cost in entropy. The system then tends to square up the modulation, which generates higher-order satellites (see *e.g.* Arons *et al.*, 1994 ▸), not observed here. This does not prevail if the magnetic carrier is a non-Kramers ion and experiences a crystalline electric field (CEF) from a low enough local symmetry, in which case its angular momentum spectrum will necessarily display at least one singlet. If this singlet is the ground state of the ion, then the magnetic order is induced by exchange interactions through mixing of the higher-energy angular momentum states with the ground singlet. In this case, a modulated magnetic structure can persist down to 0 K, as suggested by Gignoux *et al.* (1977 ▸) and observed in PrNi_2_Si_2_ (Blanco *et al.*, 1992 ▸). Ho^3+^ ions are non-Kramers ions and their site symmetry in Ho i-MAX is *m*, meaning that it is reasonable to expect a CEF singlet. Therefore, amplitude-modulated structures in this system are not particularly unusual. In addition, amplitude-modulated magnetic structures have also been observed in parent Ho i-MAX phases with aluminium (MoHoAlC), even at the lowest temperatures, as shown by neutron diffraction and muon spin rotation measurements (Tao *et al.*, 2022 ▸, 2019 ▸; Barbier *et al.*, 2022 ▸; Potashnikov *et al.*, 2021 ▸).

The microscopic origins of the stabilized magnetic phases in rare-earth (RE) i-MAX phases remain a topic of ongoing debate. As with many other RE-based intermetallic systems, crystalline electric field and Ruderman–Kittel–Kasuya–Yosida (RKKY) interactions are likely the primary factors driving the magnetic behaviour. It has also been proposed that in the Gd i-MAX phase, RE planes could be coupled via dipolar interactions, which might also be the case here (Potashnikov *et al.*, 2021 ▸). However, given the magnitude of the magnetic moments and the distance between two magnetic moments from neighbouring planes, the dipolar coupling energy would be very small (∼1 K). Additionally, it has been suggested that the incommensurate magnetic order arises from Fermi surface nesting, which would introduce the band structure morphology as a significant factor influencing the magnetic order. However, this seems unlikely because the magnetism of the (RE) i-MAX phases originates from the localized *f* electrons of the rare earth and not from the *d* conduction electrons at the Fermi level. Therefore, the origin of the particular magnetic phase presented in the current study is most probably due to a mechanism of exchange competition which is naturally present with the changing nature of the coupling as a function of distance characteristic of the RKKY interaction.

It is interesting to note that in compounds with incommensurate structures (compositional modulation or atomic displacive waves), magnetic structures have been observed in which a commensurable magnetic order, with **k** being the propagation vector, is accompanied by 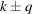 indexed reflections, where **q** is the structural modulation vector (Orlandi *et al.*, 2018 ▸; Leclercq *et al.*, 2020 ▸). In the present case, no structural incommensurability is observed at high temperature, leaving open the question of whether such a distortion (too weak to be detected) could be at the origin of the second transition.

Further studies on i-MAX phases could greatly benefit from inelastic neutron scattering experiments, which would provide crucial insights into the magnetic interactions and the CEF scheme. Combined angle-resolved photoemission spectroscopy experiments and density functional theory calculations could also provide further insights into the metallic spin polarization that might allow refinement of the exchange mechanisms, thereby shedding light on the origin of the incommensurate order. However, the current lack of single crystals presents a significant challenge to conducting these experiments. The synthesis of single crystals is therefore essential to advancing this field, though attempts made to date have not been successful. The availability of single crystals would facilitate a clear and unambiguous exploration of reciprocal space, as well as the elucidation of the intricate details of this unique structure. 

## Supplementary Material

Crystal structure: contains datablock(s) global, I. DOI: 10.1107/S2052520624011053/pz5104sup1.cif

Supporting information. DOI: 10.1107/S2052520624011053/pz5104sup2.pdf

ILL dataset: https://doi.org/10.5291/ILL-DATA.CRG-2450

CCDC reference: 2402463

## Figures and Tables

**Table 1:** Incommensurate longitudinal amplitude-modulated magnetic structure (AM1) of (Mo_2/3_Ho_1/3_)_2_GaC refined at 9 K Refinement was carried out using 137 satellites: *R*_p_ = 7.28, *wR*_p_ = 9.61, *R*(obs) = 2.66, *wR*(obs) = 3.37, *R*(all) = 3.04 and *wR*(all) = 3.40%.

Compound	(Mo_2/3_Ho_1/3_)_2_GaC at 9 K
Parent space group	*Cmcm* (No. 63)
Propagation vector	[0, 0.696 (1), 0]
Space-group preferences	SSG: basic space-group setting, orthorhombic axes **abc**
MSSG symbol	*Cmcm*.1′(0,β,0)*s*0*ss*
MSSG No.	63.1.15.11.*m*458.2
Irreducible representation	mDT2
Magnetic point group	*mmm*.1′
Unit-cell parameters (Å, °)	*a* = 9.534, *b* = 5.471, *c* = 13.482
	α = 90, β = 90, γ = 90
	
MSSG symmetry operations	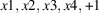
	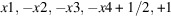
	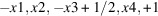
	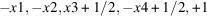
	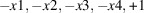
	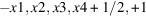
	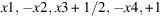
	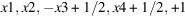
	
MSSG centering operations	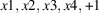
	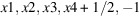
	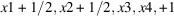
	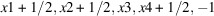
	
Positions of non-magnetic atoms	Ga1: Ga 4*c* 0.00000, 0.82300, 0.25000
	Ga2: Ga 8*g* 0.23970, 0.08730, 0.25000
	C1: C 8*e* 0.66970, 0.00000, 0.00000
	C2: C 4*a* 0.00000, 0.00000, 0.00000
	Mo1: Mo 16*h* 0.66300, 0.33030, 0.42040
	
Position of magnetic atom	Ho1: Ho 8*f* 0.00000, 0.34060, 0.38370
	
Magnetic moments: cos and sin Fourier coefficient of magnetic atoms (μ_B_) and symmetry constraints	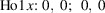
	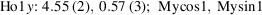
	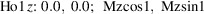

**Table 2:** Incommensurate amplitude-modulated magnetic structure model in (3+2)D of (Mo_2/3_Ho_1/3_)_2_GaC refined at 3 K Refinement was carried out using 575 satellites: *R*_p_ = 5.36, *wR*_p_ = 7.18, *R*(obs) = 4.74, *wR*(obs) = 6.19, *R*(all) = 4.74 and *wR*(all) = 6.12%.

Compound	(Mo_2/3_Ho_1/3_)_2_GaC at 3 K
Parent space group	*Cmcm* (No. 63)
MSSG symbol	*Amma*.1′ (0,β,0)00*s*0 (0,0,γ)*ss*0*s*
MSSG No.	63.2.44.59.*m*458.3
Transformation matrix to the parent phase	(0, 1, 0 | 0, 0, 1 | 1, 0, 0)
Magnetic point group	*mmm*.1′
Independent modulation vectors	**q**_1_ = (0, 0.07445, 0)
	**q**_2_ = (0, 0, 0.7034)
Irreducible representations	mDT2, SM4†
Unit-cell parameters (Å, °)	*a* = 13.5295, *b* = 9.5241, *c* = 5.4669
	α = 90, β = 90, γ = 90
	
MSSG symmetry operations	1: 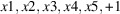
	2: 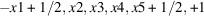
	3: 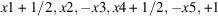
	4: 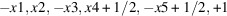
	5: 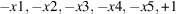
	6: 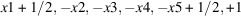
	7: 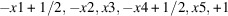
	8: 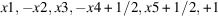
	
MSSG centering operations	1: 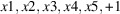
	2: 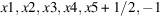
	3: 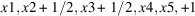
	4: 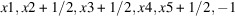
	
Positions of non-magnetic atoms	Ga1_1: Ga 4*c* 0.25000, 0.00000, 0.82300
	Ga2_1: Ga 8*g* 0.25000, 0.23970, 0.08730
	C1_1: C 8*e* 0.00000, 0.66970, 0.00000
	C2_1: C 4*a* 0.00000, 0.00000, 0.00000
	Mo_1: Mo 16*h* 0.42040, 0.66300, 0.33030
	
Position of magnetic atom	Ho_1: Ho 8*f* 0.38370, 0.00000, 0.34060
	
Wavevectors	1: 0.00000 0.07445 0.00000 1 0
	2: 0.00000 0.00000 0.70340 0 1
	3: 0.00000 0.07445 0.70340 1 1
	4: 0.00000 0.07445 −0.70340 1 −1
	
Magnetic moments: site label, axis, wavevector, cos and sin Fourier coefficient of magnetic atoms (μ_B_), and symmetry constraints	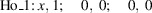
	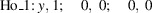
	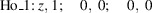
	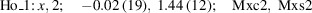
	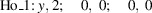
	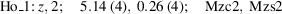
	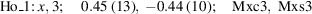
	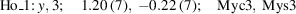
	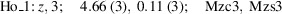
	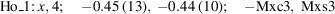
	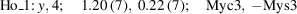
	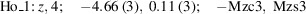

† Note that the modulation according to the irrep SM4 alone does not exist in the model; the amplitude of the modulation corresponding to wavevector 1 is zero. The wavevectors 3 and 4 correspond to a magnetic modulation along **k**_1_ + **k**_2_ and **k**_1_ − **k**_2_ propagation vectors, *i.e.*in the **k**-plane *P* of the Brillouin zone. See Section 3.6.

## Appendix A Time-inversion operator compatible with the satellite existence condition

The objective is to identify a time-inversion operator that is compatible with the satellite existence condition. To this end, consider a (3+2)D MSSG with a first modulation vector **k**_1_ = (0, *k_y_*, 0) and a second modulation vector **k**_2_ = (τ_*x*_, 0, 0). All diffraction spots can be indexed with five indices with respect to the five vectors **a***, **b***, **c***, **k**_1_, **k**_2_. The observation that only new satellites can be observed with respect to old ones can be expressed by the condition that satellite intensities are detected for (*h*, *k*, *l*, *m*, 0), (*h*, *k*, *l*, *m*, *n*) with *m* ≠ 0 and *n* ≠ 0 but not for (*h*, *k*, *l*, 0, *n*). We are therefore seeking a time-reversal operator that fulfils this condition.

The structure factor of a modulated magnetic structure is a vector and it is transformed in a similar way to a regular structure factor. This means that the symmetry operation can be described by a matrix:

where  is 3 × 3 matrix in the external (real, 3D space),  and  are *d* × 3 and *d* × *d* matrices which follow from the above equation,  are the modulation vectors *i* = 1,…, *d* and vectors  and  are translation parts. Thus, applying this operation to the structure factor leads to the following equation:

The time-inversion operator has the following form:

The *x* and *y* can be 0 or ½ and nothing else, as the product

has to be a lattice translation in (3 + 2)D superspace.

Using this operation in the equation for transformation of the structure factor leads to

For the combination *x* = ½ and *y* = 0 we have

which means for even *m* the  and the structure factor for such reflections must be zero. This means that we can observe satellites with the fourth index *m*, (*m*, 0) or (*m*, *n*), but not (0, *n*) as *m* must be odd. The time-inversion operator therefore has the form .

## References

[bb1] Arons, R. R., Loewenhaupt, M., Reif, T. & Gratz, E. (1994). *J. Phys. Condens. Matter*, **6**, 6789–6799.

[bb2] Barbier, M., Wilhelm, F., Colin, C. V., Opagiste, C., Lhotel, E., Pinek, D., Kim, Y., Braithwaite, D., Ressouche, E., Ohresser, P., Otero, E., Rogalev, A. & Ouisse, T. (2022). *Phys. Rev. B*, **105**, 174421.

[bb3] Barsoum, M. W. (2000). *Prog. Solid State Chem.* **28**, 201–281.

[bb4] Bertaut, E. F. (1968). *Acta Cryst.* A**24**, 217–231.

[bb5] Blanco, J. A., Schmitt, D. & Gómez Sal, J. C. (1992). *J. Magn. Magn. Mater.* **116**, 128–142.

[bb7] Campbell, B. J., Stokes, H. T., Tanner, D. E. & Hatch, D. M. (2006). *J. Appl. Cryst.* **39**, 607–614.

[bb8] Dahlqvist, M., Lu, J., Meshkian, R., Tao, Q., Hultman, L. & Rosen, J. (2017). *Sci. Adv.* **3**, e1700642. 10.1126/sciadv.1700642 PMC5517111 28776034

[bb9] Gallego, S. V., Perez-Mato, J. M., Elcoro, L., Tasci, E. S., Hanson, R. M., Aroyo, M. I. & Madariaga, G. (2016). *J. Appl. Cryst.* **49**, 1941–1956.

[bb10] Gignoux, D., Gomez-Sal, J. C., Lemaire, R. & de Combarieu, A. (1977). *Solid State Commun.* **21**, 637–639.

[bb11] Gordon, J. E., Dempesy, C. W. & Soller, T. (1961). *Phys. Rev.* **124**, 724–725.

[bb13] Leclercq, B., Arévalo-López, A. M., Kabbour, H., Daviero-Minaud, S., Pautrat, A., Basu, T., Colin, C. V., Das, R., David, R. & Mentré, O. (2020). *Adv. Quantum Technol.* **4**, 2000064.

[bb14] Orlandi, F., Aza, E., Bakaimi, I., Kiefer, K., Klemke, B., Zorko, A., Arčon, D., Stock, C., Tsibidis, G. D., Green, M. A., Manuel, P. & Lappas, A. (2018). *Phys. Rev. Mater.* **2**, 074407.

[bb15] Ouisse, T. & Colin, C. V. (2018). https://doi.org/10.5291/ILL-DATA.CRG-2450.

[bb16] Perez-Mato, J. M., Ribeiro, J. L., Petricek, V. & Aroyo, M. I. (2012). *J. Phys. Condens. Matter*, **24**, 163201. 10.1088/0953-8984/24/16/163201 22447842

[bb17] Petříček, V., Dušek, M. & Palatinus, L. (2014). *Z. Kristallogr. Cryst. Mater.* **229**, 345–352.

[bb18] Petříček, V., Fuksa, J. & Dušek, M. (2010). *Acta Cryst.* A**66**, 649–655. 10.1107/S0108767310030527 20962373

[bb19] Petříček, V., Palatinus, L., Plášil, J. & Dušek, M. (2023). *Z. Kristallogr. Cryst. Mater.* **238**, 271–282.

[bb20] Petruhins, A., Lu, J., Hultman, L. & Rosen, J. (2019). *Mater. Res. Lett.* **7**, 446–452.

[bb21] Pomjakushin, V., Plokhikh, I., White, J. S., Fujishiro, Y., Kanazawa, N., Tokura, Y. & Pomjakushina, E. (2023). *Phys. Rev. B*, **107**, 024410.

[bb23] Potashnikov, D., Caspi, E. N., Pesach, A., Tao, Q., Rosen, J., Sheptyakov, D., Evans, H. A., Ritter, C., Salman, Z., Bonfa, P., Ouisse, T., Barbier, M., Rivin, O. & Keren, A. (2021). *Phys. Rev. B*, **104**, 174440.

[bb24] Qureshi, N., Ruiz-Martín, M. D., Puente-Orench, I., Fernández-Díaz, M. T., Balbashov, A. M., Ivanov, V. Y., Skumryev, V. & Mukhin, A. A. (2018). *Phys. Rev. B*, **98**, 094411.

[bb25] Rodríguez-Carvajal, J. & Villain, J. (2019). *C. R. Phys.* **20**, 770–802.

[bb26] Rossat-Mignod, J. (1987). *Methods in Experimental Physics*, Vol. 23, Part C, *Neutron Scattering*, pp. 69–157. Academic Press.

[bb27] Stokes, H. T. & Campbell, B. J. (2022). *Acta Cryst.* A**78**, 364–370. 10.1107/S2053273322003898 35781417

[bb29] Tao, Q., Barbier, M., Mockute, A., Ritter, C., Salikhov, R., Wiedwald, U., Calder, S., Opagiste, C., Galera, R. M., Farle, M., Ouisse, T. & Rosen, J. (2022). *J. Phys. Condens. Matter*, **34**, 215801. 10.1088/1361-648X/ac5bcf 35259732

[bb30] Tao, Q., Lu, J., Dahlqvist, M., Mockute, A., Calder, S., Petruhins, A., Meshkian, R., Rivin, O., Potashnikov, D., Caspi, E. N., Shaked, H., Hoser, A., Opagiste, C., Galera, R. M., Salikhov, R., Wiedwald, U., Ritter, C., Wildes, A. R., Johansson, B., Hultman, L., Farle, M., Barsoum, M. W. & Rosen, J. (2019). *Chem. Mater.* **31**, 2476–2485.

