# Modulation of the modulated magnetic structure of an Ho i-MAX phase described by a magnetic (3+2)-dimensional superspace group. Corrigendum

**DOI:** 10.1107/S2052520625003361

**Published:** 2025-04-14

**Authors:** Claire V. Colin, Quanzheng Tao, Christine Opagiste, Rafik Ballou, Johanna Rosen, Thierry Ouisse, Václav Petříček

**Affiliations:** a Institut Néel Université Grenoble Alpes & CNRS Grenoble 38042 France; b Department of Physics, Chemistry and Biology (IFM) Linkopings Universitet Linköping SE-58183 Sweden; c Université Grenoble Alpes Centre national de la recherche scientifique (CNRS), Grenoble INP, Laboratoire des Matériaux et du Génie Physique (LMGP) Grenoble 38000 France; d Institute of Physics of the Czech Academy of Sciences Na Slovance 1999/2 18200Praha 8 Czechia; Universidad del País Vasco, Spain

**Keywords:** magnetic superspace groups, incommensurate structures, rare-earth i-MAX phases, neutron diffraction

## Abstract

The name of one of the authors in the article by Colin *et al.*[*Acta Cryst.* (2025), B**81**, 37–46] is corrected.

## Abstract 2

Corrigendum to *Acta Cryst.* (2025), B**81**, 37–46.

The name of the fourth author in the article by Colin *et al.* (2025 ▸) is incorrectly given as Rafik Balou. The correct spelling is Rafik Ballou, as given above.
