# Supplementary material for: Modulation of the modulated magnetic structure of an Ho i-MAX phase described by a magnetic (3+2)-dimensional superspace group
Source: Acta Crystallogr B Struct Sci Cryst Eng Mater. 2025 Jan 23;81(Pt 1):37–46. doi: 10.1107/S2052520624011053 (PMC11801704; doi:10.1107/S2052520624011053)
Supplement: Supplementary file 2 [file b-81-00037-sup2.pdf]

STRUCTURAL SCIENCE
CRYSTAL ENGINEERING
MATERIALS

Volume 81 (2025)

Supporting information for article:

Modulation of the modulated magnetic structure of an Ho i-MAX phase described by a magnetic (3+2)-dimensional superspace group

Claire V. Colin, Quanzheng Tao, Christine Opagiste, Rafik Balou, Johanna Rosen, Thierry Ouisse and Václav Petříček

S1. Crystal structure of $(Mo_{2/3}Ho_{1/3})_2GaC$ at 300K

Table S1 Crystallographic structure of $(Mo_{2/3}Ho_{1/3})_2GaC$ refined at 300K.

Space group	C m c m			
Unit cell parameters (Å)	a = 9.5472	α = 90°		
	b = 5.4735	β = 90°		
	c = 13.5021	γ = 90°		
Atomic Coordinates	Ga	0.0	0.82554(233)	0.25
	Ga	0.24040(78)	0.08835(145)	0.25
	C	0.16970(81)	0.5	0.0
	C	0.0	0.0	0.0
	Mo	0.16310(23)	0.82928(54)	0.42043(23)
	Ho	0.0	0.33956(52)	0.38401(33)
Overall B iso	0.22(3)			

Bragg R-factor: 6.6 Rp: 3.71 Rwp: 5.02

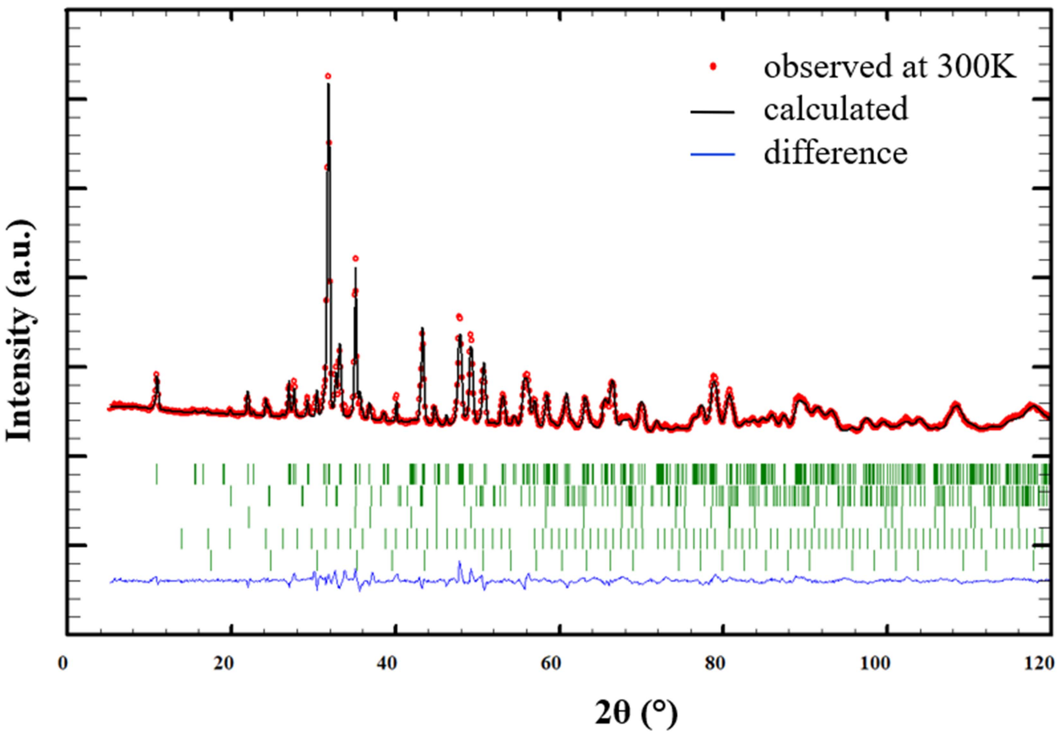

Figure S1 NPD pattern recorded at 300K with a wavelength of λ=1.28Å and Rietveld refinement of $(Mo_{2/3}Ho_{1/3})_2GaC$, including identified impurity phases. Bragg positions of identified phases (weight percentage) are indicated by green ticks, first raw: $(Mo_{2/3}Ho_{1/3})_2GaC$ (86.5(6)%), second raw: Mo_2C (6.8(2)%), third raw: C (0.30(2)%), fourth raw: Ho_2O_3 (4.6(3)%), fifth raw: $HoGa_3$ (1.8(2)%).

S2. Exploration of orthorhombic MSSG

A trial-and-error search via ISODISTORT exploration of (3+2)D MSSGs with (0, k_y, 0) and (τ_x, 0, 0) yielded a vast number of possibilities. Therefore, in order to narrow down the search, it was necessary to constrain it.

All diffraction spots can be indexed with five indices with respect to the five vectors ***a****, ***b****, ***c****, $\boldsymbol{k_1}$, $\boldsymbol{k_2}$. This implies that from the superspace approach, a set of reflections (h, k, l, m, n) can be derived. The observation that only new satellites can be observed with respect to old ones can be expressed by the condition that satellite intensities are detected for (h, k, l, m, 0), (h, k, l, m, n) with m≠0 and n≠0 but not for (h, k, l, 0, n) (see Figure 5b). As it is demonstrated in Appendix A, this dictates the form of the operator that combines time inversion with a translation in the internal subspace of the superspace.

In the *Amma* setting, the most symmetrical solution compatible with the detected satellite reflection is the choice of modulation vectors $(0,\beta,0)$ and $(0,0,\gamma)$ with the time inversion operator

$\left(x_1, x_2, x_3, x_4, x_5 + 1/2 \middle| -1\right)$. This gives 16 different MSSG that were systematically tested. The results are presented in Table S2. The 4 groups in bold are those derived from the symmetry of the AM1 phase (hypothesis 1 discussed in the main text). Subgroups 6 and 7 give the best solutions.

Table S2 Orthorhombic MSSG refinements

Name	MSSG	GOF	Rp	Rwp	R(All)
Subgroup-1	Amma.1'(0,b1,0)0000(0,0,g2)000s	19.97	20.78	28.48	24.45
Subgroup-2	Amma.1'(0,b1,0)0000(0,0,g2)0s0s	16.34	17.09	23.28	22.02
Subgroup-3	Amma.1'(0,b1,0)00s0(0,0,g2)000s	21.27	22.45	30.33	29.09
Subgroup-4	Amma.1'(0,b1,0)00s0(0,0,g2)0s0s	17.56	17.64	25.02	22.29
Subgroup-5	Amma.1'(0,b1,0)0000(0,0,g2)s00s	10.98	10.42	15.66	8.54
**Subgroup-6**	**Amma.1'(0,b1,0)0000(0,0,g2)ss0s**	**5.04**	**5.36**	**7.18**	**4.75**
**Subgroup-7**	**Amma.1'(0,b1,0)00s0(0,0,g2)ss0s**	**5.04**	**5.36**	**7.18**	**4.75**
Subgroup-8	Amma.1'(0,b1,0)00s0(0,0,g2)s00s	11.16	10.29	15.92	7.49
Subgroup-9	Amma.1'(0,b1,0)s000(0,0,g2)000s	11.51	11.21	16.42	9.45
Subgroup-10	Amma.1'(0,b1,0)s000(0,0,g2)0s0s	8.21	8.91	11.70	10.02
Subgroup-11	Amma.1'(0,b1,0)s0s0(0,0,g2)000s	11.51	11.21	16.42	9.45
Subgroup-12	Amma.1'(0,b1,0)s0s0(0,0,g2)0s0s	8.24	8.86	11.74	9.29
Subgroup-13	Amma.1'(0,b1,0)s000(0,0,g2)s00s	16.89	16.96	24.09	22.11
**Subgroup-14**	**Amma.1'(0,b1,0)s000(0,0,g2)ss0s**	**11.08**	**11.40**	**15.79**	**12.38**
**Subgroup-15**	**Amma.1'(0,b1,0)s0s0(0,0,g2)ss0s**	**11.08**	**11.40**	**15.79**	**11.18**
Subgroup-16	Amma.1'(0,b1,0)s0s0(0,0,g2)s00s	17.96	17.39	25.62	19.24

The subgroup-7 model is presented in the main text in **Error! Reference source not found.**. The subgroup-6 model is presented in Table S3. Although this model allows a refinement of the data comparable to model 7, the refined moments for Holmium are not physical. As can be seen in Figure S2 , the maximum value is 15.5 μ_B which is considerably larger than the theoretical value expected for Ho.

Table S3 Incommensurate amplitude-modulated magnetic structure model at (3+2)D of $(Mo_{2/3}Ho_{1/3})_2GaC$ refined at 3K with the subgroup-6 model.

Compound	$(Mo_{2/3}Ho_{1/3})_2GaC$ – 3K
Parent space group	*Cmcm* (N. 63)
MSSG symbol	*Amma.1’ (0,b_1,0)0000 (0,0,g_2)ss0s*
MSSG number	63.2.44.57.m458.3
Transformation matrix to the parent phase	(0,1,0 | 0,0,1 | 1,0,0)
Magnetic point group	*mmm.1'*
Independent modulation vectors	q_1= (0, 0.07445, 0)

	q_2 = (0, 0, 0.7034)
Irreducible representations	mDT2, SM1
Unit cell parameters (Å)	a = 13.5295 α = 90°<br>b = 9.5241 β = 90°<br>c = 5.4669 γ = 90°
MSSG symmetry operations	1 x1,x2,x3,x4,x5,+1<br>2 -x1+1/2,-x2,x3,-x4,x5,+1<br>3 -x1,x2,-x3,x4,-x5+1/2,+1<br>4 x1+1/2,-x2,-x3,-x4,-x5+1/2,+1<br>5 -x1,-x2,-x3,-x4,-x5,+1<br>6 x1+1/2,x2,-x3,x4,-x5,+1<br>7 x1,-x2,x3,-x4,x5+1/2,+1<br>8 -x1+1/2,x2,x3,x4,x5+1/2,+1
Centering	1 x1,x2,x3,x4,x5,+1<br>2 x1,x2,x3,x4,x5+1/2,-1<br>3 x1,x2+1/2,x3+1/2,x4,x5,+1<br>4 x1,x2+1/2,x3+1/2,x4,x5+1/2,-1
Positions of non-magnetic atoms	Ga1_1 Ga 4 c 0.25000 0.00000 0.82300<br>Ga2_1 Ga 8 g 0.25000 0.23970 0.08730<br>C1_1 C 8 e 0.00000 0.66970 0.00000<br>C2_1 C 4 a 0.00000 0.00000 0.00000<br>Mo_1 Mo 16 h 0.42040 0.66300 0.33030
Positions of magnetic atom	Ho_1 Ho 8 f 0.38370 0.00000 0.34060
Wave vectors	1 0.00000 0.07445 0.00000 1 0<br>2 0.00000 0.00000 0.70340 0 1<br>3 0.00000 0.07445 0.70340 1 1<br>4 0.00000 0.07445 -0.70340 1 -1
Magnetic moments: site label, wave-vector, axis, cos and sin Fourier coefficient of magnetic atoms (μ_B) and symmetry constraints	Ho_1 1 x 0.00000 0.00000 0 0<br>Ho_1 1 y 0.00000 0.00000 0 0<br>Ho_1 1 z 0.00000 0.00000 0 0<br>Ho_1 2 x 0.00000 -1.55(16) Mxc2 Mxs2<br>Ho_1 2 y 0.00000 0.00000 0 0<br>Ho_1 2 z -5.52(6) -0.28(5) Mzc2 Mzs2<br>Ho_1 3 x 0.48(18) -0.48(14) Mxc3 Mxs3<br>Ho_1 3 y 1.28(10) -0.24(10) Myc3 Mys3<br>Ho_1 3 z 5.00(5) 0.12(4) Mzc3 Mzs3<br>Ho_1 4 x 0.48(18) 0.48(14) Mxc3 -Mxs3

Ho_1	4	-1.28(10)	-0.24(10)	-Myc3 Mys3
Ho_1	4 z	5.00(5)	-0.12(4)	Mzc3 -Mzs3

Refinement done on 575 satellites: Rp= 5.36 wRp= 7.18 R(obs)= 4.74 wR(obs)= 6.19 R(all)= 4.74 wR(all)= 6.12

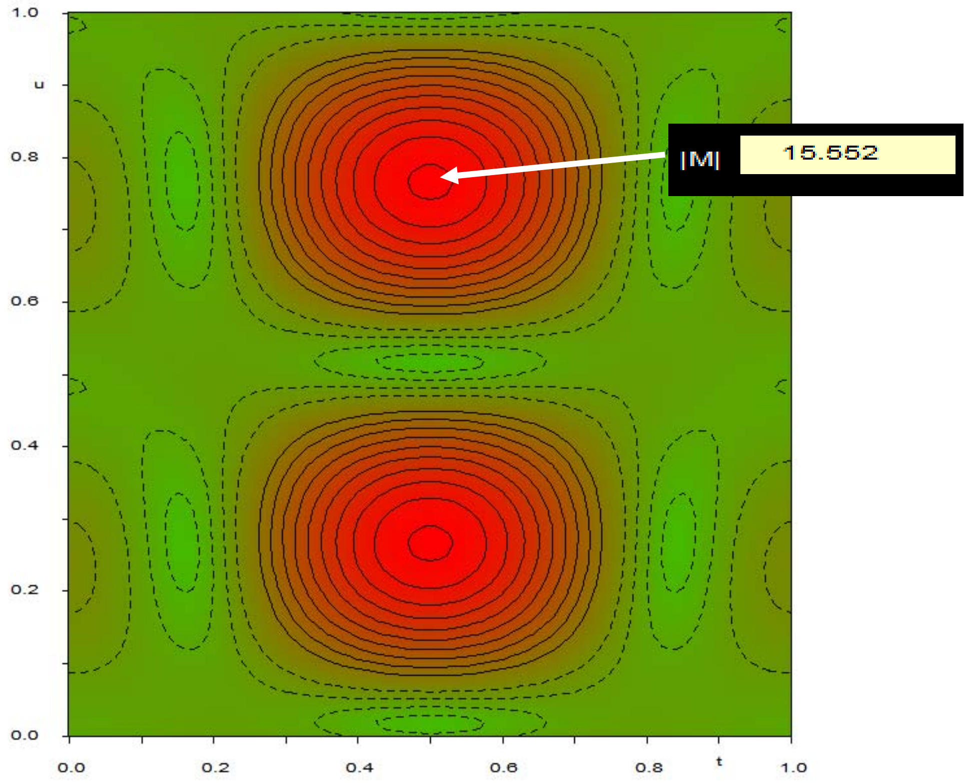

Figure S2 Contour plot of the absolute value of the magnetic moment of Ho (in μ_B) as a function of the internal coordinates t and u refined with the Subgroup-6 model (*Amma.1' (0,b$_1$,0)0000 (0,0,g$_2$)ss0s*). The maximum value is 15.5 μ_B which is considerably larger than the theoretical value expected for Ho.

S3. Exploration of monoclinic MSSG

It is possible to index all magnetic peaks considering two modulation vectors (0, k_y, 0) and (τ_x, k_y, 0). This combination is not compatible with the orthorhombic symmetry, indeed, the vector (τ_x, k_y, 0) (k-plane P) lower the symmetry to a monoclinic $P2_1/m.1'$ symmetry. A systematic exploration of all 4 potential MSSG calculated with ISODISTORT was conducted. The results of the refinements are

presented in Table S4. The results obtained for monoclinic models are markedly inferior to those obtained when considering the orthorhombic symmetry (see S2).

Table S4 Monoclinic MSSG refinements:

Name	MSSG	GOF	Rp	Rwp	R(All)
Subgroup-1	P2_1/m.1'(a1,b1,0)00s(a2,b2,0)000	19.10	19.42	27.23	30.72
Subgroup-2	P2_1/m.1'(a1,b1,0)0ss(a2,b2,0)000	6.43	7.22	9.17	9.85
Subgroup-3	P2_1/m.1'(a1,b1,0)0s0(a2,b2,0)00s	8.62	9.21	12.29	14.41
Subgroup-4	P2_1/m.1'(a1,b1,0)0s0(a2,b2,0)00s	9.99	11.03	14.25	14.45

S4. Exploration of orthorhombic MSSG in (3+3)D

An orthorhombic model can also be constructed by considering three modulation vectors: ((0, k_y, 0), (τ_x, k_y, 0) and (-τ_x, k_y, 0), where the value of k_y is identical in the three components. A systematic exploration of all potential MSSG was conducted. The results of the refinements are presented in Table S5. The best model was obtained combining the mDT2 and mP2 Irreps, however the refined moments for Holmium obtained for this model are not physical. As can be seen in Figure S2, the maximum value is 15.65 μ_B which is considerably larger than the theoretical value expected for Ho. Furthermore, the indicators of the fit quality obtained for this models are markedly inferior to those obtained when considering the (3+2)D orthorhombic symmetry.

Table S5 Orthorhombic (3+3)D refinements:

Name - Irreps	GOF	Rp	Rwp	R(All)
Subgroup-mP1mDT1	19.78	20.71	28.21	25.88
Subgroup-mP1mDT2	9.62	10.64	13.70	14.51
Subgroup-mP1mDT3	16.44	16.68	23.45	21.78
Subgroup-mP1mDT4	15.92	16.88	22.68	22.85
Subgroup-mP2mDT1	11.38	10.96	16.23	11.59
**Subgroup-mP2mDT2**	**5.08**	**5.50**	**7.24**	**8.06**
Subgroup-mP2mDT3	10.80	10.17	15.40	10.53
Subgroup-mP2mDT4	7.87	8.63	11.21	10.89

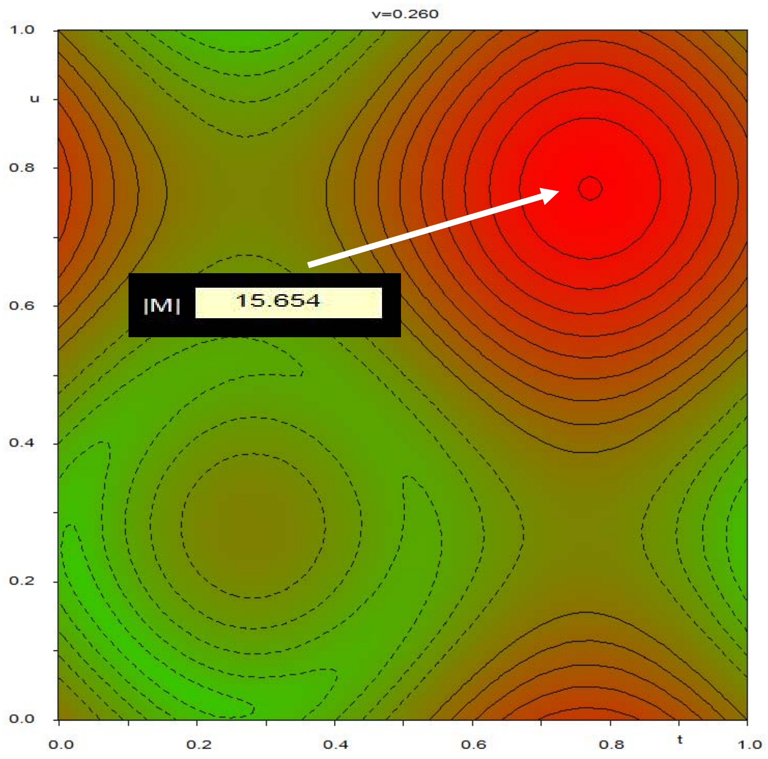

Figure S3 Contour plot of the absolute value of the magnetic moment of Ho (in μ_B) as a function of the internal coordinates t and u refined with the Subgroup- mP2mDT2. The maximum value is 15.6 μ_B which is considerably larger than the theoretical value expected for Ho.
